# Urokinase-type plasminogen activator receptor (uPAR) as a promising new imaging target: potential clinical applications

**DOI:** 10.1111/cpf.12037

**Published:** 2013-04-03

**Authors:** Morten Persson, Andreas Kjaer

**Affiliations:** 1 Department of Clinical Physiology, Nuclear Medicine and PET, Center for Diagnostic Investigations, RigshospitaletCopenhagen, Denmark; 2 Cluster for Molecular Imaging, Faculty of Health Sciences, University of CopenhagenCopenhagen, Denmark; 3 The Danish-Chinese Center for Proteases and CancerCopenhagen, Denmark

**Keywords:** cancer, human, mouse, MRI, oncology, optical Imaging, pet, proteolysis, SPECT, translational, uPAR/uPA

## Abstract

Urokinase-type plasminogen activator receptor (uPAR) has been shown to be of special importance during cancer invasion and metastasis. However, currently, tissue samples are needed for measurement of uPAR expression limiting the potential as a clinical routine. Therefore, non-invasive methods are needed. In line with this, uPAR has recently been identified as a very promising imaging target candidate. uPAR consists of three domains attached to the cell membrane via a glycosylphosphatidylinositol (GPI) anchor and binds it natural ligand uPA with high affinity to localize plasminogen activation at the cell surface. Due to the importance of uPAR in cancer invasion and metastasis, a number of high-affinity ligands have been identified during the last decades. These ligands have recently been used as starting point for the development of a number of ligands for imaging of uPAR using various imaging modalities such as optical imaging, magnetic resonance imaging, single photon emission computer tomography (SPECT) and positron emission topography (PET). In this review, we will discuss recent advances in the development of uPAR-targeted imaging ligands according to imaging modality. In addition, we will discuss the potential future clinical application for uPAR imaging as a new imaging biomarker.

## Introduction

**Figure 1:**
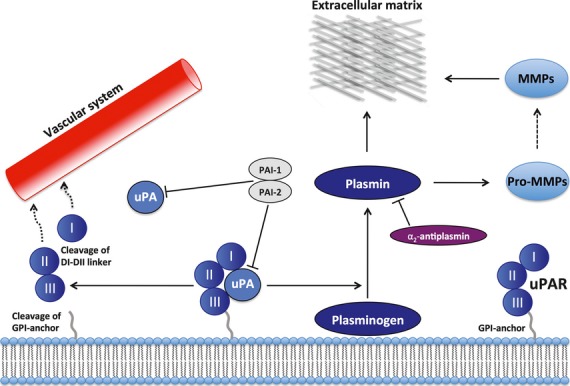
Schematic overview of the uPA/uPAR system. The uPAR is anchored to the plasma membrane and binds specifically to uPA. uPA can catalyse the process from plasminogen to plasmin. Two plasminogen activator inhibitors, PAI-1, PAI-2, however, inhibit this cleavage reaction. Plasmin can then either directly degrade ECM or activate other proteases such as metalloproteases (MMP), thereby promoting cancer spread. Plasmin is inhibited by alpha-2 antiplasmin. Membrane-anchored uPAR can finally be cleaved, resulting in secretion of uPAR domains into the vascular system.

The plasminogen activator (PA) system (Figure 1) plays an important role in various physiological processes involving tissue remodelling (Blasi & Sidenius, 2010). In addition, the PA system has also been shown to have a key role in the pathogenesis of vascular diseases, including atherosclerosis, thromboembolic disorders and stroke (Nicholl *et al*., 2006). However, it was the observation of urokinase in urine of patients with cancer, first reported in 1960 (Riggenbach & von KAULLA, 1960) that initiated the extensive research into the PA system. The PA system consists of the serine protease urokinase-type plasminogen (uPA), its glycosylphosphatidylinositol (GPI)-anchored cell membrane receptor (uPAR), the substrate plasminogen and the plasminogen activator inhibitors PAI-1 and PAI-2 (Figure 1). In 1988, a study in patients with breast cancer was the first to demonstrate uPA is a prognostic marker for survival in cancer (Duffy *et al*., 1988). At the same time, the receptor for uPA (uPAR) was identified (Vassalli *et al*., 1985). uPAR consists of three domains (D1, D2 and D3) and is attached to the cell membrane via a GPI anchor and promotes pericellular proteolysis by binding uPA (Ploug *et al*., 1991a; Ploug *et al*. 1991b). The association between uPAR and cancer was recognized in 1991 (Ossowski *et al*., 1991). Since then, extensive literature has described uPAR to be of special importance for cancer invasion and metastasis (Casslen *et al*., 1991; Pyke *et al*., 1993; Ganesh *et al*., 1994; Dano *et al*., 2005; Dass *et al*., 2008; Jacobsen & Ploug, 2008).

Using various biochemical assays, studies have shown that uPAR is expressed both in malignant epithelial cells and non-malignant stromal cells in the tumour microenvironment, for example, macrophages and neutrophils (Pyke *et al*., 1994; Hildenbrand *et al*., 2000), whereas the expression level in normal homoeostatic tissue is limited. Moreover, uPAR can be cleaved from the membrane (Ploug *et al*., 1992), and higher levels of uPAR and/or various uPAR forms in the blood have been reported in a number of cancers compared with healthy controls. The level of uPAR is also a prognostic marker for metastatic diseases and poor prognosis (Ganesh *et al*., 1994; Miyake *et al*., 1999b; Stephens *et al*., 1999; Foekens *et al*., 2000; Riisbro *et al*., 2002; de Bock & Wang, 2004; Jacobsen & Ploug, 2008; Rasch *et al*., 2008; Lomholt *et al*., 2010). However, measurement of plasma levels of uPAR will always only be an indirect indicator for the expression level in the tumour. Moreover, the lack of correlation between tumour tissue uPAR expression and the level of secreted forms (e.g. D1 + D2D3) further complicate the information achievable (de Witte *et al*., 2001). This is perhaps also the reason for the lack of routine clinical use of plasma uPAR measurements. It seems that localized measurements in the tumour and in the local microenvironment are necessary for optimal uPAR-based diagnostic and prognostic information. Indeed, detailed uPAR immunohistochemistry studies on tumour tissue have revealed that an increasing uPAR expression is present at the very front of the invasive tumour, and uPAR is therefore considered a potential local marker for invasive cancers (Pyke *et al*., 1994; Dublin *et al*., 2000; Jacobsen & Ploug, 2008; Van & Van, 2009; Alpízar-Alpízar *et al*., 2012). In line with this, uPAR has recently been identified as a very promising imaging target (Yang *et al*., 2011). In that study, uPAR was found to be in the same category as other well-known imaging targets such as HER2, integrins and EGFR. The development of a clinical imaging agent for the detection of uPAR-expressing tissue could potentially have relevance both in patient stratification and therapy monitoring, because a number of uPAR-targeted therapeutic ligands have been reported in the literature. They have primarily aimed for the inhibition of the uPA–uPAR interaction and have been based on small molecules, peptides, proteins, cytotoxins, anti-gene therapy and therapeutic radionuclides (Kriegbaum *et al*., 2011).

The advantages of non-invasive imaging over conventional biopsy-based techniques are obvious. Due to the heterogeneity of cancer (Hanahan & Weinberg, 2011), including uPAR expression, any biopsy may or may not represent the true tumour expression level of uPAR. Moreover, cleaved forms of uPAR in the blood have shown not to correlate with the amount of uPAR present in the tumour tissue. Indeed, previously no correlation was found between uPAR levels in serum and tumour cytosols of patients breast cancer (de Witte *et al*., 2001). This observation could suggest that the increased circulating levels found in patients with cancer are not solely the result of an increased amount of uPAR being shed from the primary tumour tissue, but perhaps from micrometastases and/or inflammatory tissue in response to the cancer.

In this article, we review studies published within non-invasive molecular imaging of uPAR, categorized according to imaging modality. In addition, we will discuss potential future clinical applications of uPAR imaging.

## Optical imaging

To date, only a few studies have reported the use of optical imaging ligands for uPAR (Table 1). A uPAR-targeting ligand consisting of 11-amino acid alkyl-modified peptide inserted into the outer layer of a stealth liposome nanoparticle has been described (Wang *et al*., 2009). The authors characterized the nanoparticle, containing plasmid DNA, in uPAR-positive DU145 human prostate cancer cells and found a high and specific uptake with improved transfection level, compared with uPAR-negative HEK293 cells. A Cy5.5-labelled monoclonal anti-uPAR antibody was used in a proof-of-concept study to visualize human mammary cancer MDA-MB-231 xenograft in mice (Dullin 2009). A correlation between fluorescence signal and tumour size was found. Binding specificity was analysed both *in vitro* using uPAR-negative BT474 cells and *in vivo* using non-uPAR binding Cy5.5-IgG1 antibody. In both control experiments, no fluorescence was detected.

**Table 1:** Overview of uPAR ligands for optical and magnetic resonance imaging

Imaging modality	Ligand	Name	*In vitro* data	*In vivo* data	Reference
Optical	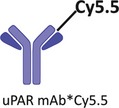	uPAR mAb^*^Cy5.5	Yes	Yes	Dullin, 2009
	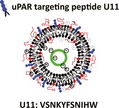	U11	Yes	No	Wang *et al*. (2009)
	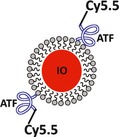	mATF-Cy5.5-IO	Yes	Yes	Yang *et al*. (2009,b)
	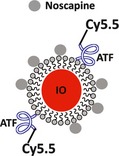	hATF-Cy5.5-IO-Nos	Yes	No	Abdalla *et al*. (2011)
	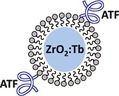	Zro2:Tb-hATF	Yes	No	Liu *et al*. (2012)
MR	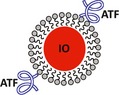	mATF-Cy5.5-IO	Yes	Yes	Yang *et al*. (2009,b)
	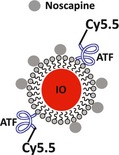	hATF-Cy5.5-IO-Nos	Yes	No	Abdalla *et al*. (2011)

Two other studies used optical imaging to develop and validate a paramagnetic nanoparticle using magnetic resonance imaging (MRI) (Yang *et al*., 2009,b). These studies will be described later in the section below on MRI. Recently, a report of an amine-functionalized ZrO_2_-Tb nanoparticle with amino-terminal fragment (ATF) of human uPA covalently attached was published (Liu *et al*., 2012). The specific recognition of the ATF-coupled nanoparticle was only tested *in vitro* using confocal laser scanning microscopy. A high signal intensity was found in uPAR-positive H1299 non-small cell lung cancer cells compared with uPAR-negative human embryo lung fibroblast (HELF) cells that were used as control. No *in vivo* data on this nanoparticle have been reported although this could be interesting to pursue.

The relatively limited number of reports of ligands for uPAR imaging using optical imaging may reflect the somewhat limited clinical potential of this technology. Due to the short penetration of light in tissue, only surface exposed tissue can be imaged using this technology. Therefore, the only potential clinical utility of uPAR-targeted optical imaging ligands seems to be within image-guide surgery (Keereweer *et al*., 2011; Mieog *et al*., 2011). Image-guide surgery is a technology where the surgeon intra-operatively is guided by imaging of the tumour. Near-infrared fluorescent ligands are mostly used because their wavelength is invisible to the human eye and therefore does not cause any interference with visualization of the operating field. Total removal of tumour tissue during curative-intended cancer surgery is pivotal. However, the correct delineation of tumour tissue from normal tissue is often difficult. As uPAR is expressed along the invasive front of multiple tumours, uPAR-targeted ligands for optical imaging could potentially be used as a new method for establishing valid resection margins during surgery, which potentially could reduce the number of patients with postoperative recurrences of cancer.

## Magnetic resonance imaging

Contrary to ligands for optical imaging, MRI-based ligands possess higher translational potential. The development of dedicated small-animal MRI systems (James & Gambhir, 2012) has made the transition from preclinical to clinical testing easier. MRI has the advantage of an outstanding anatomical resolution, whereas the drawback is the low sensitivity, typical in the millimolar range (James & Gambhir, 2012).

The development of uPAR-targeted ligands for MRI has focused entirely on paramagnetic iron oxide nanoparticles. One research group published two studies using uPAR-specific nanoparticles containing iron oxide for MRI in pancreatic cancer (Yang *et al*., 2009a)and breast cancer (Yang *et al*., 2009b). In both studies, the mice-based amino-terminal fragments (mice ATF) of uPA were conjugated to the nanoparticle and used as human uPAR-specific ligand. High and specific uptake was apparently reported compared with a uPAR-negative cell lines of murine origin, generating a strong MRI contrast detectable by 3 tesla MRI. The results presented were, however, based on the assumption of no species specificity between mouse ATF and human uPAR. There has, however, been strong evidence of this species specificity (Lin *et al*., 2010) showing that mouse ATF has a significantly reduced binding to human uPAR and therefore this assumption is at least controversial. Finally, did one preliminary study, report the use of an ATF-coated nanoparticle for uPAR-targeted delivery of noscapine, a plant alkaloid-binding tubulin with known anticancer effect in human PC-3 prostate cancer cells *in vitro* (Abdalla *et al*., 2011). The nanoparticles were coated with Cy5.5 and loaded with iron oxide, thus enabling the nanoparticles to be tracked by both optical imaging and MRI. A six-fold stronger inhibitory effect of PC-3 cells was found for the nanoparticle compared with free noscapine, thus an indication of the specific uPAR-directed internalization of the nanoparticle.

The potential clinical utility of uPAR-targeted MRI-ligands is promising at first sight. With the high resolution, a clear identification of invasive cancers seems possible. However, due to the limited sensitivity, the amount of ligand necessary to obtain sufficient signal (mM range) seems to be a challenge as toxicological aspects needs to be considered at this dose levels, in contrast to SPECT and PET, where the amount of ligand necessary is much lower (typical in the nanomolar range).

## SPECT imaging

SPECT imaging provides the necessary sensitivity for detection of small micrometastatic lesions and invasive cancer and therefore has a potential broad clinical utility. Moreover, the high availability of gamma cameras, low-cost and easy radiochemistry, at least when considering the use 99mTc, makes SPECT imaging of uPAR highly attractive. Despite this, only two studies have reported on the development of SPECT-based ligands for uPAR (Armstrong *et al*., 2009; Liu *et al*., 2009). In one study, synthesis and characterization of a SPECT-imaging based human uPAR-targeting ^111^in-labelled peptide was described (Liu *et al*., 2009). They used a dimeric linear peptide (AE120) (Ploug *et al*., 2001), with C-terminal DOTA conjugation. *In vitro* receptor studies demonstrated an IC_50_ of 240 ± 125 nM. *In vivo* biodistribution of the peptide was performed in MDA-MB-231 tumour–bearing mice, with a tumour uptake of 0.53 ± 0.11%ID per g, 4 h postinjection. This resulted in a tumour/blood and tumour/muscle ratio of 4.2 and 9.4, respectively. A scramble control peptide was also investigated and a significant reduced tumour uptake (0.36 ± 0.11%ID per g) was found 4 h postinjection. However, the relatively low tumour uptake and reduced in vitro binding, compared with the unconjugated peptide (IC_50_ 10-20 nM) reported in that study, could most likely be attributed to the use of C-terminal DOTA conjugation. Detailed studies of the uPAR-peptide interaction have revealed limited space for any modification in the C-terminal of the peptide (AE120) without the possibility of losing binding affinity (Ploug *et al*., 2001; Llinas *et al*., 2005; Huai *et al*., 2006).

Another approach was taken by a group in Canada; they synthesized and labelled a cyclic human uPAR–targeted peptide with 99mTc (Armstrong *et al*., 2009). The applicability of this ligand is, however, also questionable as 99mTc in this case is complex-bound to a large heterocyclic tridentate chelator, which was covalently bound to lysine in the cyclic peptide mimicking uPA. The corresponding lysine amino acid in uPA has been shown to be tightly buried in the ligand interface of the uPA–uPAR complex thus excluding any modification without losing affinity (Lin *et al*., 2010). In line with this, they reported a 30-fold reduction in affinity between the unconjugated (which had identical affinity as uPA) and chelator-conjugated peptide(Table 2).

**Table 2:** Overview of uPAR ligands for SPECT and PET imaging

Imaging-modality	Ligand	Name	*In vitro*-data	*In vivo*-data	Reference
SPECT	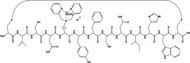		Yes	No	Armstrong *et al*. (2009)
	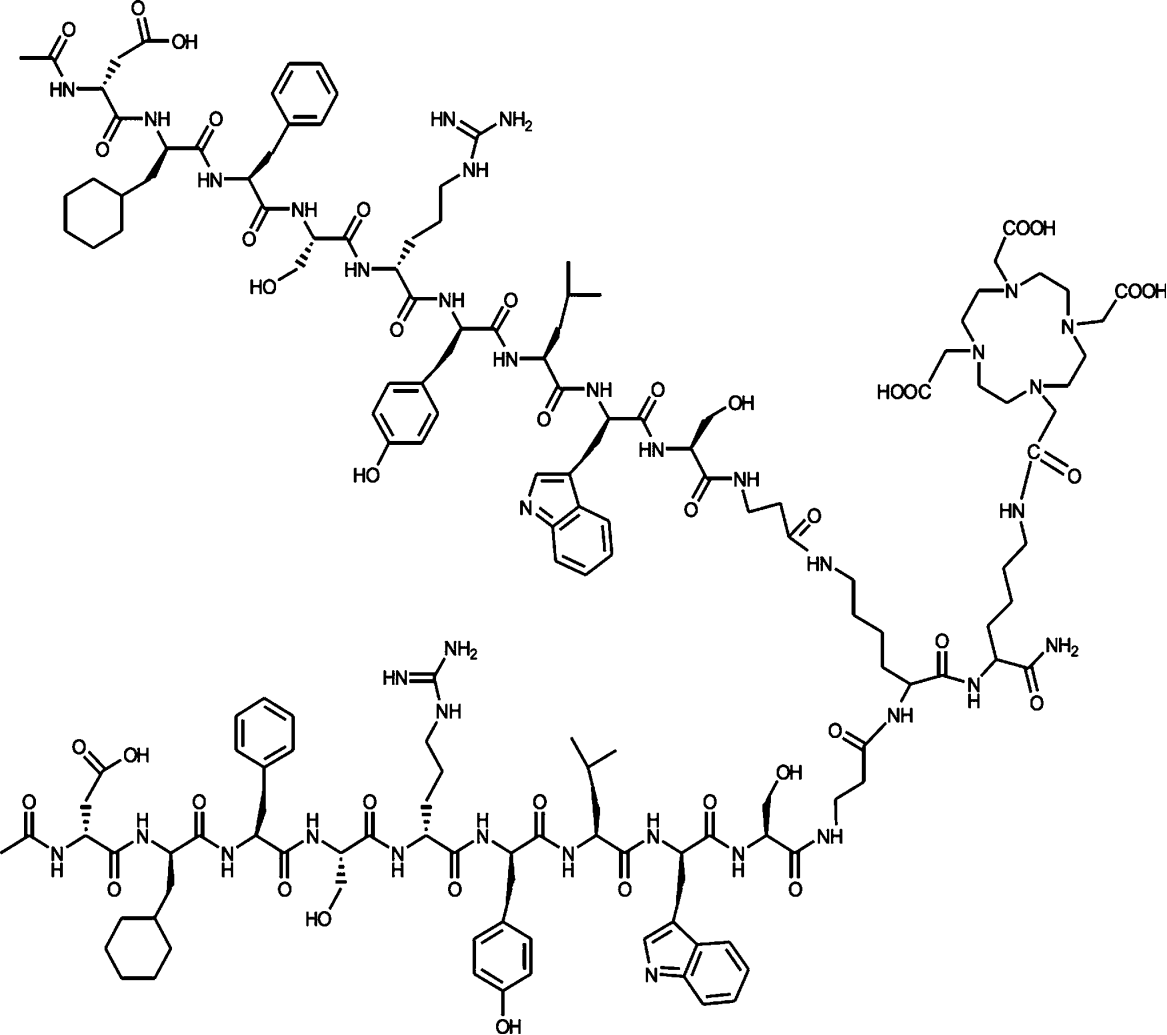	(Nac-cD-Cha-F-dS-dR-Y-L-W-S-βAla)2-K-K(DOTA)-NH2	Yes	Yes	Liu *et al*. (2009)
PET	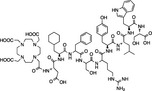	DOTA-AE105	Yes	Yes	Li *et al*. (2008), Persson *et al*. (2012,b)
	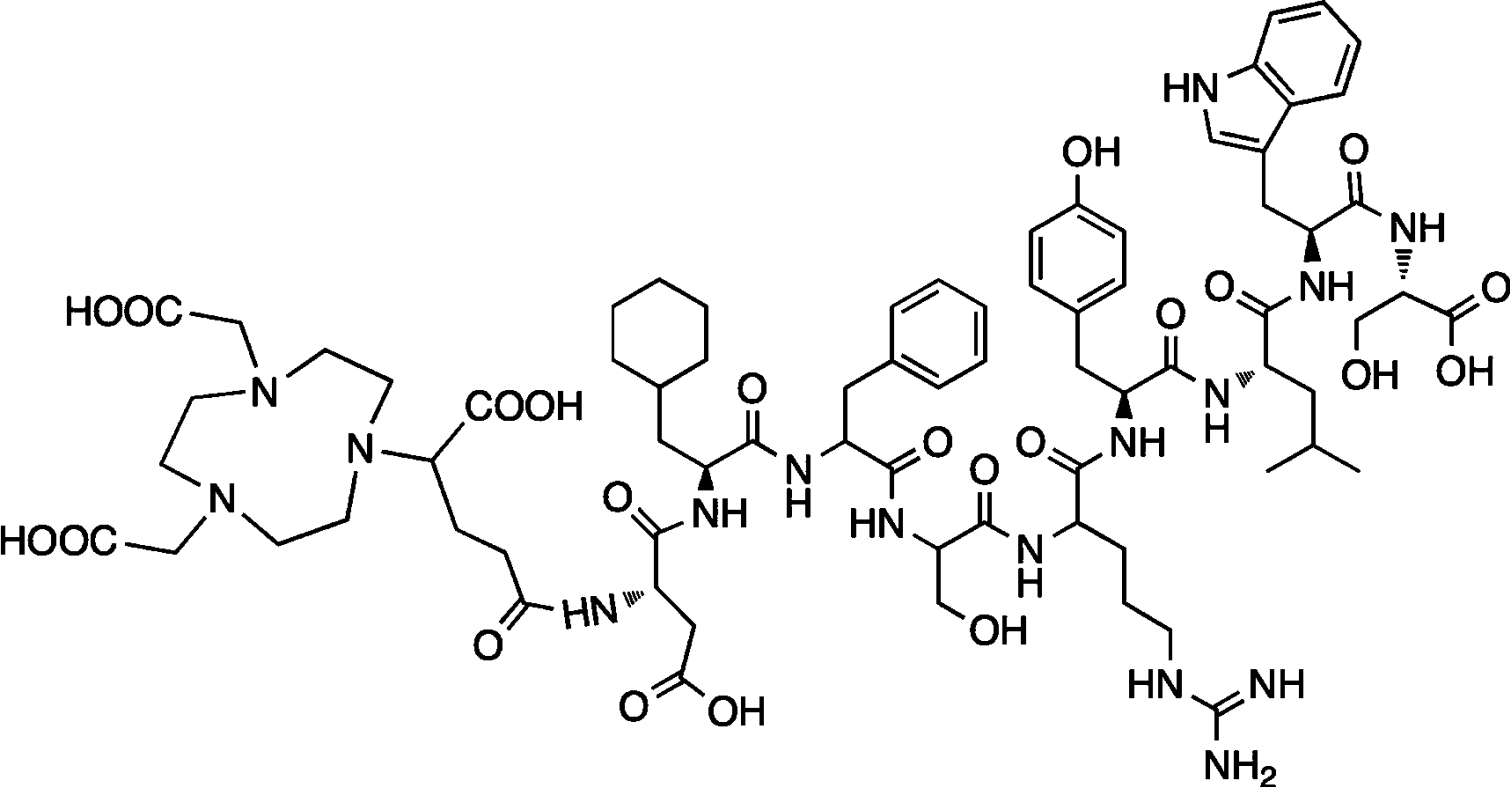	NODAGA-AE105	Yes	Yes	Persson *et al*. (2012b)
	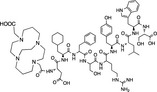	CB-TE2A-AE105	Yes	Yes	Persson (2012) Unpublished
	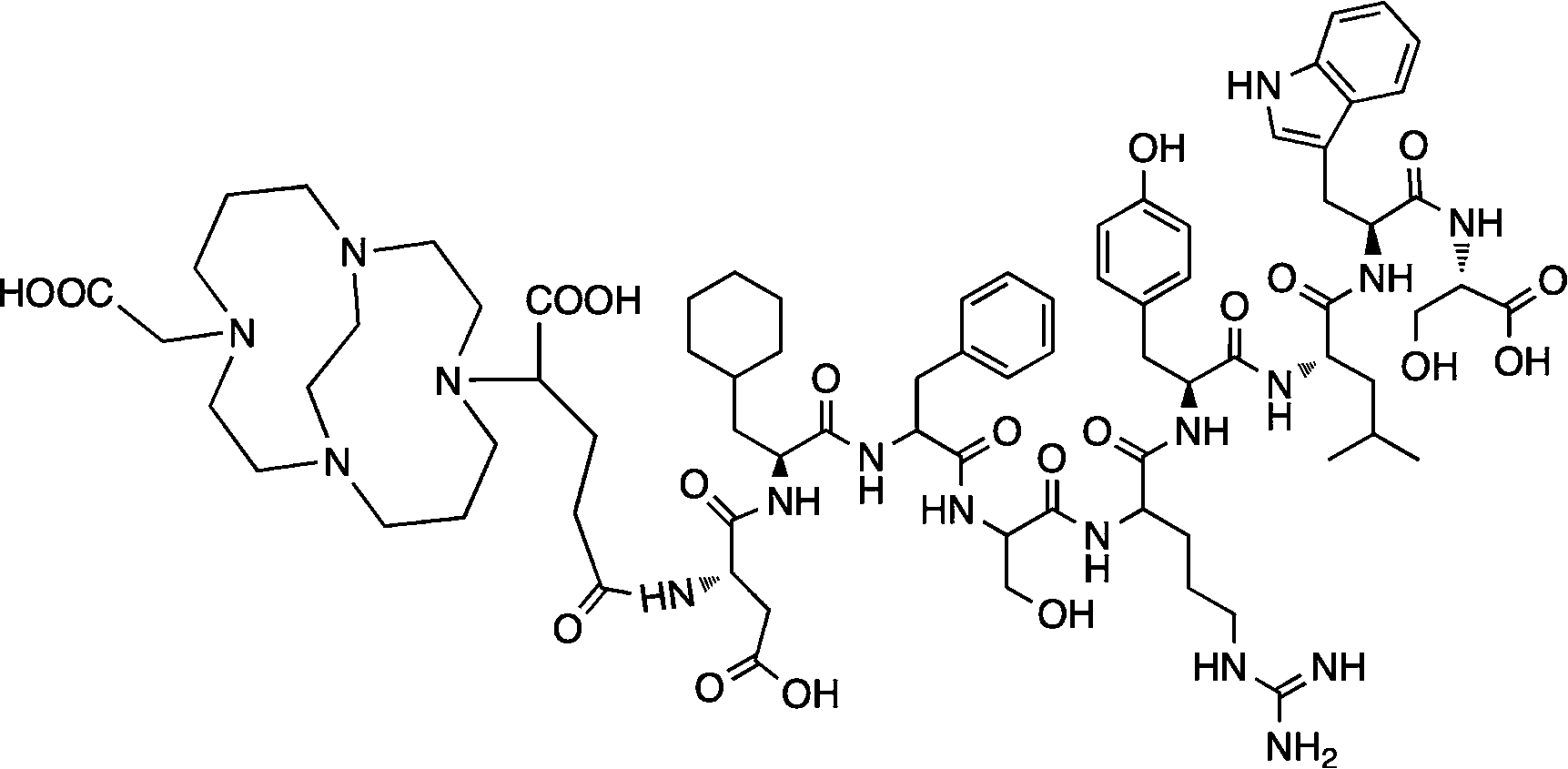	CB-TE2A-PA-AE105	Yes	Yes	Persson,-2012-Unpublished

Based on the high sensitivity of SPECT imaging, there would be a high potential for clinical use of a uPAR-targeted ligand, for example, for tumour risk stratification, therapy planning and monitoring. However, one important drawback is the lack of truly quantitative data using SPECT imaging. As described earlier, uPAR is also expressed in normal tissue. Together with the fact that the increase in uPAR expression typically follows tumour grade as in prostate cancer (Miyake *et al*., 1999a), the ability to obtain truly quantitative data, for example, for establishing an empirical cut-off value seems to be important in a clinical setting.

## PET imaging

The use of PET imaging seems to have the advantages necessary for optimal imaging of uPAR in a clinical setting. High sensitivity together with truly quantitative data suggests that PET imaging might be the best imaging modality for uPAR. In line with this, PET is also the area where most imaging studies have been performed. In the first uPAR PET proof-of-concept study (Li *et al*., 2008), a monomer version of AE120, denoted AE105 (Ploug *et al*., 2001), conjugated with the metal chelator DOTA in the N-terminal and labelled with ^64^Cu were used. MicroPET imaging of mice bearing uPAR-positive U87MG human glioblastoma and uPAR-negative MDA-MB-435 human breast cancer xenograft was used to illustrate the ability to specifically detect human uPAR. *In vitro* affinity (IC_50_) of the DOTA-conjugated peptide towards uPAR was found to be 130 nM, compared with 16 nM for the peptide without DOTA, as assessed by surface plasmon resonance (Li *et al*., 2008). A high accumulation in the uPAR-positive U87MG xenograft tumour (10.8 ± 1.5%ID per g) compared with the uPAR-negative MDA-MB-435 xenograft tumour (1.2 ± 0.6%ID per g) was found 4.5 h after injection. The specificity of the tracer was further validated by comparing the uptake of a non-binding variant of the peptide in the uPAR-positive U87MG xenograft and by performing a blocking experiment using excessive predosing of non-labelled peptide. The accumulation of the non-binding peptide in 3 mice bearing U87MG was 2.2 ± 0.5%ID per g 4.5 h postinjection and 3.7 ± 1.3%ID per g 4.5 h after bolus injection of a blocking dose of un-labelled peptide, both significantly reduced, thus illustrating the specificity of ^64^Cu-DOTA-AE105 for non-invasive PET imaging of uPAR.

**Figure 2:**
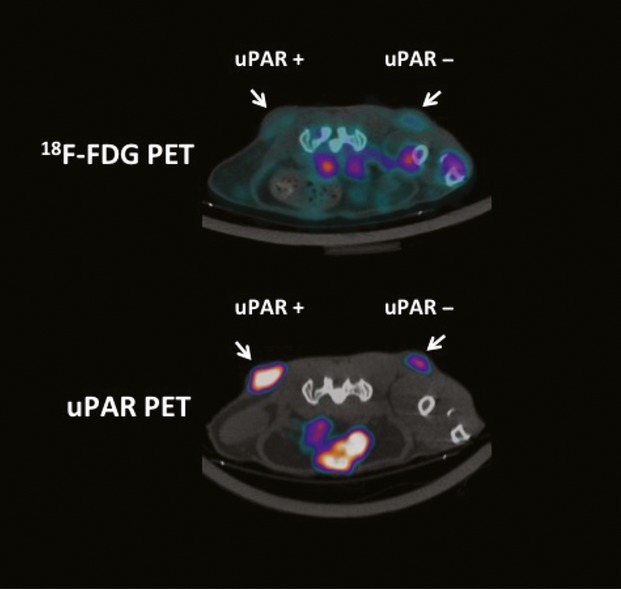
Transverse images of a mouse inoculated with a high and low uPAR expression human tumour xenograft subcutaneously after a PET scan with ^18^F-FDG (Top) and ^64^Cu-DOTA-AE105 (uPAR PET) (Bottom), 1 h postinjection.

In our group, the focus has also been on the small peptide AE105 in our efforts to bring a uPAR-targeted PET ligand into clinical use. Importantly, we reported a correlation between tumour uptake of ^64^Cu-DOTA-AE105 on microPET images of human tumour xenografts and uPAR expression level in the tumour tissue (Persson *et al*., 2012)(Figure 2). The ability to perform a non-invasive quantification of uPAR expression in different tissues as demonstrated by us could potentially enable use of an empirically based cut-off value, for example, differentiating normal tissue from tumour tissue and aggressive cancers from indolent tumours, in clinical settings.

We have also investigated the use of different metal-binding chelators and different isotope, in our effort to develop an optimal uPAR PET ligand for clinical use. Our results using ^64^Cu-DOTA-AE105 (Persson *et al*., 2012) revealed a relatively high accumulation of ^64^Cu in the liver, a known site for ^64^Cu accumulation and a well-established indirect marker of instability of ^64^Cu-based ligands in rodents (Bass *et al*., 2000; Boswell *et al*., 2004). In line with this and based on the fast tumour uptake observed in our study, we hypothesized that the use of ^68^Ga instead of ^64^Cu could maintain tumour uptake and reduce the non-specific uptake in non-target tissue, especially the liver. Furthermore, the half-life of ^68^Ga more resembles the biological half-life of our peptide-based ligand, and as ^68^Ga is a generator-based radionuclide, this could make our ligand more widely used in PET centres. The results of using ^68^Ga showed a significant reduction in liver uptake as expected for both ^68^Ga-DOTA-AE105 and ^68^Ga-NODAGA-AE105 (Persson *et al*., 2012b). However, this reduction was also accompanied by a reduction in tumour uptake and a lower tumour-to-kidney ratio, compared with ^64^Cu-DOTA-AE105. The overall results were an improved 5.0-fold tumour-to-liver ratio, but also a 4.4-fold and 2.4-fold reduction in absolute tumour uptake, for ^68^Ga-DOTA-AE105 and ^68^Ga-NODAGA-AE105, respectively. A higher muscle uptake was also observed for ^68^Ga-based ligands, resulting in poor PET images because of reduced tumour-to-background ratio.

Based on these findings, we choose to take a different approach, instead of using a different radionuclide; we went back to ^64^Cu, but with the use of new and improved metal chelator based on cross-bridge cyclam (Weisman *et al*., 1990; Anderson *et al*., 2008). These chelators were N-conjugated to our AE105 uPAR-targeting peptide. The first head-to-head comparison study between the two different cross-bridge cyclam chelators resulted in a somewhat surprising result (Persson M, Hosseini M, Madsen J, Jensen KJ, Kjaer A, Ploug M, unpublished data). A significant higher tumour-to-liver uptake was found for ^64^Cu-CB-TE2A-PA-AE105 compared with both ^64^Cu-CB-TE2A-AE105 and ^64^Cu-DOTA-AE105 at both 1 h and 22 h postinjection thus indicating superior *in vivo* performance. At 22 h postinjection, a significant higher tumour uptake was found for ^64^Cu-DOTA-AE105, however, due to the known instability of ^64^Cu-DOTA complex, and the fact that any free ^64^Cu seems to have a relatively high accumulation in tumour tissue as recently reported (*Jørgensen et al*., Nuclear Medicine & Biology, Accepted, Jan. 2013) the higher tumour uptake at 22 h postinjection must be interpreted with caution.

Overall, the most promising PET ligand for uPAR imaging still seems to be ^64^Cu-DOTA-AE105 based on the fast and high tumour uptake and the close correlation between uPAR expression and uptake in tumour tissue, despite the stability issues in mice. However, this instability of ^64^Cu-DOTA in mice seems to be of less importance in humans as recently illustrated in a first-in-humans study using ^64^Cu-DOTA-TATE in patients with neuroendocrine tumour, where low liver uptake was found and with high tumour accumulation (Pfeifer *et al*., 2012). This confirms that our tumour mouse models are not ideal models of humans. So despite not perfect in preclinical use, ^64^Cu-DOTA-AE105 currently seems to be the uPAR PET ligand with the highest potential for fast translation into clinical testing.

## Perspective

Greatest translational potential for imaging of uPAR lies within the use of radionuclide-based imaging, where PET imaging of patients’ with cancer seems to be highly promising due to the much higher sensitivity and quantitative nature compared with SPECT. Here, the first human uPAR PET study should confirm the promising preclinical data reported, that is, that one or more of these PET tracers accumulate in uPAR-positive cancer tissues in humans. Moreover, the first human uPAR PET study should also investigate whether the tumour-specific uptake contains any patient relevant information such as tumour grade, overall survival, time to metastatic development or chemotherapy resistance.

Besides uPAR PET imaging in patients with cancer, increasing evidence is also accumulating describing uPAR to be important in, for example, arteriosclerosis. Accordingly, once established for use in patients with cancer, uPAR PET imaging may prove valuable also in other disease entities.

## Conflict of interest

The authors have no conflicts of interest.
